# *Salmonella* from Farm to Table: Isolation, Characterization, and Antimicrobial Resistance of *Salmonella* from Commercial Broiler Supply Chain and Its Environment

**DOI:** 10.1155/2021/3987111

**Published:** 2021-10-06

**Authors:** M. Nasim Sohail, D. Rathnamma, S. Chandra Priya, S. Isloor, H. D. Naryanaswamy, S. Wilfred Ruban, B. M. Veeregowda

**Affiliations:** ^1^Department of Para-Clinic, Faculty of Veterinary Sciences, Afghanistan National Agricultural Sciences and Technology University (ANASTU), Kandahar-0093, Afghanistan; ^2^- Department of Veterinary Microbiology, Veterinary College, Hebbal, 560024, Bengaluru, India; ^3^- Karnataka Veterinary Animal and Fisheries Sciences University, Nandinagar, 585401, Bidar, India; ^4^Department of Livestock Products and Technology, Veterinary College, Hebbal, Bengaluru 560024, India

## Abstract

Antimicrobial resistance (AMR) in poultry production chain is one of the major food safety concerns due to indiscriminate usage of antibiotics and the presence of pathogens such as *Salmonella* which causes infections in various stages of production. In the present study, 182 samples were collected from commercial broiler supply chain, viz., three hatcheries (*n* = 29), three commercial broiler farms (CBF; *n* = 99), and three retail meat shops (RMS; *n* = 54), and used for isolation and identification of *Salmonella* using three different selective agar media and a selective enrichment medium followed by PCR confirmation targeting the *hilA* gene. The overall prevalence of *Salmonella* was 47/182 (25.82%), and a significantly higher (*P* < 0.05) prevalence was observed in retail meat shops (46.29%), CBF (19.19%), and hatcheries (10.34%). Comparison of three agar media for isolation of Salmonella revealed that all the media were equally selective. However, PCR amplification of *hilA* gene fragment was significantly higher (*P* < 0.01) in selective enrichment culture tetrathionate brilliant green bile broth (TTB) as compared to all solid (agar-based) media. Susceptibility pattern against most frequently used antibiotics revealed that 100% of the isolates were resistant to at least one antibiotic. High resistance was observed for doxycycline (94.34%), followed by cefpodoxime (84.91%), ciprofloxacin (72.64%), gentamicin (65.09%), enrofloxacin (61.32%), colistin sulphate (40.42%), amikacin (34.91%), ampicillin (33.96%), neomycin (33.02), cefotaxime (30.19%), ceftazidime (29.25%), trimethoprim-sulfamethoxazole (23.58%), amoxicillin+clavulanic acid (21.70%), and chloramphenicol (12.26%); 16.98% of the isolates were ex-tended spectrum *β*-lactamase (ESBL) producers, and 76.41% were multidrug resistant (MDR). MDR Salmonella were significantly higher (*P* < 0.01) in RMS (91.66%) followed by CBF (82.75%), whereas no MDR isolates were present in the isolates from hatcheries. The results indicated a higher prevalence of Salmonella and AMR for commonly used antibiotics in the complete broiler supply chain, especially RMS and CBF. Also, this study idicated that TTB enrichment followed by PCR and colony PCR was found to be rapid, specific and time-saving method.

## 1. Introduction

Worldwide, broiler production is an intensive system which comprises of several entities, including the breeding sector, the hatcheries, and the production sector. Any of these stages of production could serve as the source for pathogenic microorganisms. Among the various pathogens, *Salmonella* is recognized as one of the most important zoonotic and foodborne pathogens in broiler production chain [1]. Human outbreaks of foodborne illness caused by *Salmonella* worldwide have implicated that contaminated poultry and its products as the major source [2]. Contamination of poultry and its products by *Salmonella* may occur at any stage of broiler production chain [3, 4], and hence, delineating the potential risk of *Salmonella* at these various stages is imperative from the perspective of consumers and public health.

In addition to causing foodborne illness, *Salmonella* from broiler production chain have been found to be resistant to antibiotics [5] [6], and this is a growing concern requiring attention for mitigating antimicrobial resistance (AMR). Indiscriminate use of antimicrobials in poultry flocks especially for disease prevention, treatment, and growth promotion is considered as the main reason for development of AMR in bacteria that represent a risk to human health [7]. Even though it has been documented that poultry and its products are the major source of *Salmonella* causing illness in humans, their role in the transmission of AMR pathogens and antibiotic resistance genes (ARGs) into the food chain is only gaining prominence.

Control of *Salmonella* in integrated poultry supply chain is very complicated, as it involves investigation of all the inputs as well as environmental samples. Therefore, this study was designed to investigate the prevalence and distribution of *Salmonella* in complete commercial broiler supply chain (hatchery, commercial broiler farm, and retail meat shops) and to determine the AMR pattern of these isolates from commercial broiler supply chain in Karnataka, India. One of the major obstacles in detection of *Salmonella* is the isolation, and hence, the success rate is mainly dependent on the sampling procedure combined with use of selective and sensitive culture method [8]. The conventional or standard methods for isolation of *Salmonella* generally take 4–7 days and are therefore laborious, require substantial manpower, and are of low in sensitivity. Several studies have reported varying levels of *Salmonella* recovery using different selective media following enrichment [9]. In this study, we compared Xylose Lysine Deoxycholate (XLD) agar, Xylose Lysine Tergitol 4 (XLT4), and Brilliant Green Agar with Phosphates (BGA) for isolation of *Salmonella*. Presumptive colonies as per colony morphology were then subjected to colony PCR targeting the *hilA* (hyperinvasive locus A) gene.

## 2. Materials and Methods

### 2.1. Location of the Study

The study location was in and around Bengaluru, India. Samples were collected from three hatcheries (Dibbur, Nelamanglla, and Doddaballapura Taluks, of Bengaluru Rural district), three commercial broiler farms (Malur, Mulbagal, and Kolar Taluks of Kolar district), and three retail meat shops (two from Ganga Nagar, Bengaluru; one from Rahmat Nagar, Bengaluru). In this study, samples were collected from birds that belonged to the same batch along the production cycle (hatchery, CBF and RMS).

### 2.2. Collection of Samples

The details of various samples collected from each source have been depicted in Table 1. Samples were collected at single point of time from hatcheries and retail meat shops. In CBF, samples were collected three times in a single crop cycle, viz., day 0, days 18-20, and days 35-42. Samples were collected in sterile plastic containers and transported under refrigerated condition in ice packs to the laboratory and were processed on the same day.

### 2.3. Isolation of *Salmonella*

*Salmonella* were isolated using previously described standard methods [10]. In brief, all the samples were preenriched in Buffered Peptone Water (BPW) followed by incubation at 37°C for 18-24 hrs. After preenrichment, selective enrichment was done by transferring one mL of preenriched broth culture in to a tube containing nine mL of TTB and incubated at 37 to 42°C for 24 to 48 hrs. After selective enrichment, 1.5 mL of enriched TTB broth culture was subjected to DNA extraction by boiling method [11]. One *μ*L of this DNA was used for PCR targeting the *hilA* gene as described previously [12]. To study the selectivity of the three commonly used agar media, one loopful of culture was streaked onto XLD, XLT4, and BGA plates. The plates were incubated at 37°C for 24-48 hrs and observed for *Salmonella* colonies (black centred red colonies on XLD and XLT4 and reddish pink/pinkish white colonies on BGA). Four colonies per sample were selected and subjected to biochemical tests. Further, colony PCR targeting the *hilA* gene was performed, along with *Salmonella* Typhimurium (ATCC® 14028™) as a positive control and *Escherichia coli* (ATCC® 25922™) as a negative control, in addition to nuclease free water (NFW) as no template control (NTC). For colony PCR, directly each colony was just touched with the tip of sterile microtip and was mixed to a PCR mixture as a template, in a total volume of 25 *μ*L. The PCR was performed with initial denaturation at 94°C for 5 min and 30 cycles of 94°C for 30 sec, 65°C for 1 min, 72°C for 1 min, and final extension of 72°C for 10 min using the published primers 5′CGGAACGTTATTTGCGCCATGCTGAGGTAG3 ′ and 5′-GCATGGATCCCCGCCGGCGAGATTGTG-3′ [12].

### 2.4. Detection of *Salmonella enterica*

All *Salmonella* isolates obtained in the study were subjected for *Salmonella enterica* species specific PCR, targeting the *iroB* gene. As a template, one *μ*L of DNA extracted was added to a PCR mixture, with a total volume of 25 *μ*L. The PCR was performed with initial denaturation at 94°C for 5 min followed by 30 cycles of 94°C for 40 sec., 55°C for 40 sec., 72°C for 40 sec., and final extension of 72°C for 10 min using the published primers 5′-TGCGTATTCTGTTTGTCGGTCC-3′ and 5′-TACGTTCCCACCATTCTTCCC-3′ [13].

### 2.5. Phenotypic Characterization of AMR in *Salmonella*

*Salmonella* isolates were subjected for antimicrobial susceptibility testing based on disc diffusion assay using gentamicin (GEN = 10 *μ*g), amikacin (AK = 30 *μ*g), neomycin (N = 10 *μ*g), ciprofloxacin (CIP = 5 *μ*g), enrofloxacin (EX = 5 *μ*g), doxycycline (DO = 30 *μ*g), trimethoprim-sulfamethoxazole (COT = 25(23.75/1.25 *μ*g)), chloramphenicol (C = 30 *μ*g), ampicillin (AMP = 10 *μ*g), amoxicillin+clavulanic acid (AMC = 20/10 *μ*g), cefotaxime (CTX = 30 *μ*g), ceftazidime (CAZ = 30 *μ*g), cefpodoxime (CPD = 10 *μ*g), cefotaxime+clavulanic acid (CEC = 30/10 *μ*g), ceftazidime+clavulanic acid (CAC = 30/10 *μ*g), and cefpodoxime+clavulanic acid (CCL = 10/05 *μ*g). Interpretation of the results was carried out as per European Committee on Antimicrobial Susceptibility Testing (EUCAST) [14] and Clinical and Laboratory Standard Institute (CLSI) wherever the EUCAST breakpoints were not available. There are no breakpoints for doxycycline, instead the breakpoints for tetracycline were used. The *Salmonella* isolates were classified as susceptible or resistant to the antimicrobial agents used.

### 2.6. Screening of the *Salmonella* Isolates for ESBL

*Salmonella* isolates were subjected for ESBL screening, and the results were interpreted as per CLSI [15]. Resistance to at least one of the three antibiotics (cefotaxime (≤ 27 mm), ceftazidime (≤ 22 mm), and cefpodoxime (≤ 17 mm)) was considered as positive in the screening test for possible ESBL production. Isolates of *Salmonella* that were considered to be positive for ESBL production by the screening test were subjected to the phenotypic confirmatory test [16] using disks of cefotaxime + clavulanic acid (CEC = 30/10 *μ*g), ceftazidime + clavulanic acid (CAC = 30/10 *μ*g), and cefpodoxime + clavulanic acid (CCL = 10/5 *μ*g). An increase in the zone diameter by ≥5 mm containing cephalosporin with clavulanic acid over the disks containing cephalosporin alone for any one of the groups indicated ESBL production.

### 2.7. Determination of Minimum Inhibitory Concentration (MIC) of Colistin

**Figure 1:**
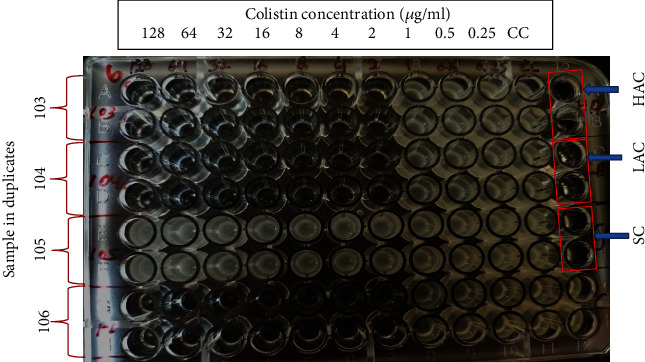
Determination of MIC of colistin sulphate for the *Salmonella* isolates: *Salmonella* isolates 103, 104, 105, and 106. CC: culture control; HAC: highest antibiotic control; LAC: lowest antibiotic control; SC: sterility control wells.

Standard broth microdilution technique [15] was employed for assessing the MIC of colistin sulphate (CS), using cation-adjusted Mueller-Hinton broth (CAMHB, HiMedia). The test was performed in untreated polystyrene flat bottom 96 well plates. Different concentrations of colistin sulphate ranging from 0.25 to 128 mg/L were prepared in CAMHB in plates and were inoculated with the isolates, and the plates were incubated at 37°C for 18 hrs. The MIC end point was determined as the lowest concentration of colistin that completely inhibited visible growth (Figure 1).

Sterility control (SC, CAMHB only), highest antibiotic control (HAC): CAMHB +128 *μ*g/mL of colistin, and lowest antibiotic control (LAC): CAMHB +0.25 *μ*g/mL were maintained in duplicates in each plate. *Escherichia coli* (ATCC® 25922™) was used as quality control for each batch of screening. The cut-off value was interpreted as per the EUCAST breakpoint (2 *μ*g/mL for colistin sulphate), and the OD value of 0.1 at 600 nm was considered as the cut-off value for conversion into numeric data to determine the resistance or sensitivity of the isolates.

## 3. Results

### 3.1. Isolation of *Salmonella* Using Three Different Media and Colony PCR

**Figure 2:**
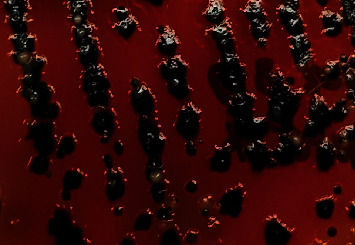
Black centred irregular translucent colonies with red coloration of the media in XLD.

**Figure 3:**
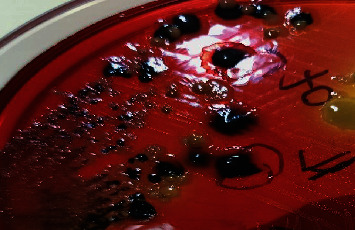
Black centred irregular translucent colonies with red coloration of the media in XLT4.

**Figure 4:**
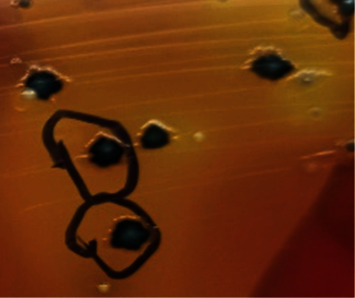
Black centred opaque colonies without red coloration of the media in XLD.

**Figure 5:**
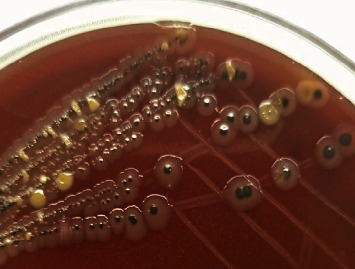
Black centred opaque colonies without red coloration of the media in XLT4.

**Figure 6:**
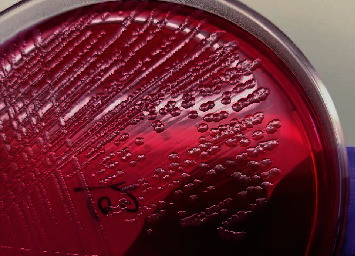
Typical pink or pinkish white Salmonella colonies with red coloration of the media in BGA.

**Figure 7:**
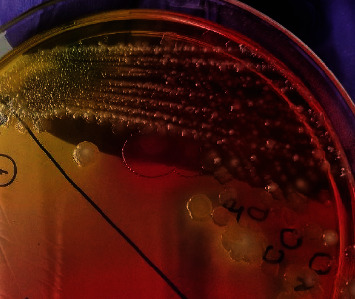
Yellow color colonies without red coloration of the media in BGA.

All the 182 samples collected from hatcheries, CBF, and RMS after selective enrichment were plated onto three different selective media XLD, XLT4, and BGA, and samples that revealed black centred colonies with red coloration of the media on XLD and XLT4 and pink or pinkish white colonies on BGA were considered to be presumptive for *Salmonella*. Of the 182 samples tested, 143 (78.57%), 115 (63.18%), and 106 (58.24%) samples were showing suspicious colonies on XLD, XLT4, and BGA, respectively (*P* < 0.002). In the present study, colonies that appeared irregularly shaped, translucent, and black centred with red coloration of the media (i.e., nonlactose fermentative) in XLD and XLT4 were confirmed to be *Salmonella* by both biochemical tests and PCR (Figures 2 and 3), whereas colonies that appeared regular, opaque, and black centred without red coloration in XLD and XLT4 (Figures 4 and 5) were negative for *Salmonella* by both biochemical tests and PCR. Similarly, in BGA, typical pink or pinkish white colonies with red coloration of the media were confirmed as *Salmonella* by biochemical tests and PCR (Figure 6), while yellow color colonies without red coloration of the media on BGA were confirmed as negative for *Salmonella* by biochemical tests and PCR (Figure 7).

**Figure 8:**
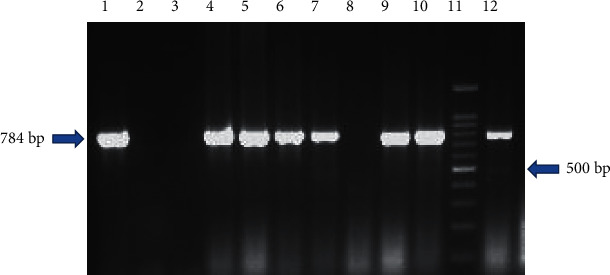
Agarose gel electrophoresis of colony PCR *hilA* gene amplicon. Lane 1: positive control (*Salmonella* Typhimurium ATCC 14028); lane 2: negative control (*Escherichia coli* ATCC® 25922™); lane 3: no template control (NTC); lanes 4, 5, 6, 7, 9, 10, and 12: samples positive for *Salmonella* spp.; lane 8: sample negative for *Salmonella* spp.; lane 11: 100 bp DNA ladder.

All the presumptive or suspected colonies were subjected to biochemical test and PCR targeting the *hilA* gene, and it was observed that only 29 (15.93%) each from XLD and BGA and 28 (15.38%) from XLT4 agar were positive and confirmed as *Salmonella* (Figure 8). A total of 106 *Salmonella* isolates were obtained in this study and were used for further characterization.

**Figure 9:**
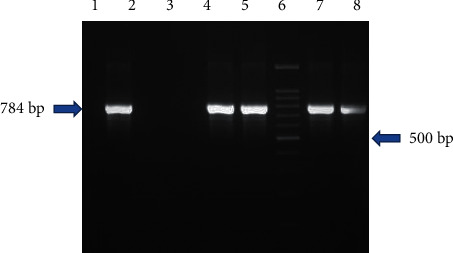
Agarose gel electrophoresis of *hilA* gene amplicon from TTB culture. Lane 1: positive control (*Salmonella* Typhimurium ATCC 14028); lane 2: negative control (*Escherichia coli* ATCC® 25922™); lane3: NTC; lanes 4, 5, 7, and 8: samples positive for *Salmonella* spp.; lane 6: 100 bp DNA ladder.

In the present study, it was found that enrichment of all the 182 samples in TTB followed by DNA extraction and PCR targeting the *hilA* gene, 52 samples (28.57%) were found positive for *Salmonella* as compared to 29 (15.93%) samples each in XLD and BGA and 28 (15.38%) samples in XLT4 (*P* < 0.001), indicating that enrichment followed by direct DNA extraction and PCR was more sensitive compared to enrichment followed by selective plating and colony PCR (Table 2 and Figure 9).

### 3.2. Prevalence of *Salmonella*

In the present study, the overall prevalence of *Salmonella* in broiler supply chain was 25.82% (47/182). A significantly higher (*P* < 0.001) prevalence was observed in RMS (25/54; 46.29% (25/54)), followed by CBF (19/99; 19.19%) and hatcheries (3/29; 10.34%). In the hatcheries, samples from incubator air tunnels, egg tray of hatchers, and yolk sac from dead chicks were positive for *Salmonella* (Table 3). With respect to CBF, the prevalence of *Salmonella* was highest from samples collected from day 0 (10/33; 30.30%), followed by days 35-42 (6/33; 18.18%) and days 18-20 (3/33; 9.09%). In addition, in CBF, the prevalence in samples collected from internal environment of the farms was 40% (6/15) as compared to samples collected from external environment (3/9; 33.33%), followed by faecal swabs (5/15: 33.33%), water sample from nipples/drinkers, and feed sample from different feeders (2/15; 13.33%) and feed bags (1/15; 6.66%) (Table 4). However, none of the samples collected from water tank were positive. In RMS, *Salmonella* was recovered from ileal and cecal contents, chicken carcasses, meat rinsing water, and knife swabs. On the other hand, none of the swabs from surface of cutting/chopping board sample were positive (Table 5).

### 3.3. Detection of *Salmonella enterica*

**Figure 10:**
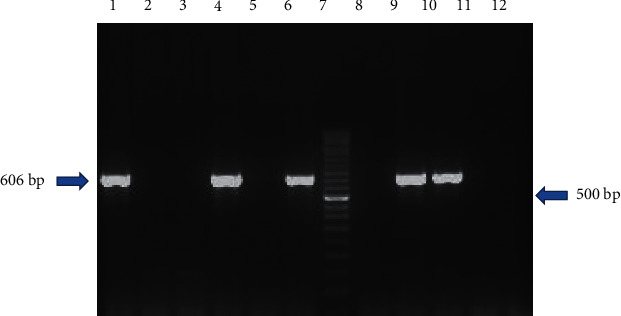
Agarose gel electrophoresis of *iroB* gene amplicon for *Salmonella enterica*. Lane 1: positive control (*Salmonella* Typhimurium ATCC 14028); lane 2: negative control (*Escherichia coli* ATCC® 25922™); lane 3: NTC; lanes 4, 6, 9, and 10: samples positive for *Salmonella* spp.; lanes 5,8, 11, and 12: samples negative for *Salmonella* spp.; lane 7: 50 bp DNA ladder.

**Figure 11:**
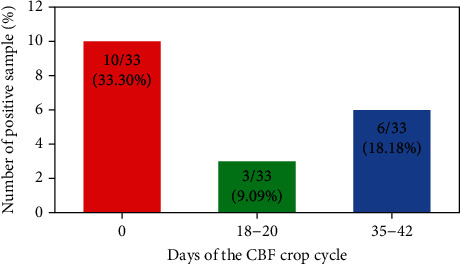
Detection of *Salmonella* in CBF at different points of crop cycle.

Among 106 *Salmonella* isolates, 69 (65.09%) were confirmed as *Salmonella enterica* by species-specific PCR targeting *iroB* gene. The prevalence of *Salmonella enterica* was found to be higher in isolates from RMS (27/36; 75%) followed by hatcheries (8/12; 66.66%) and CBF samples (34/58; 58.62%) (Table 6 and Figures 10 and Figure 11).

### 3.4. Antimicrobial Susceptibility Pattern

Antimicrobial susceptibility testing of *Salmonella* isolates from hatcheries (*n* = 12) revealed that all isolates were resistant to cefpodoxime followed by ciprofloxacin and doxycycline (58.33%) and 8.33% to both gentamycin and cefotaxime, whereas none of the isolates were found resistant to amikacin, neomycin, enrofloxacin, trimethoprim-sulfamethoxazole, chloramphenicol, ampicillin and amoxicillin + clavulanic acid, ceftazidime, and colistin sulphate. Among the CBF isolates (*n* = 58), highest resistance was observed against doxycycline (100%) and least to chloramphenicol (3.45%) (Table 7).

Comparison of antimicrobial susceptibility pattern of CBF isolates obtained during crop cycles from day 0 to days 35-42 revealed highest resistance to doxycycline and least to chloramphenicol, whereas on days 18-20, 100% resistance was observed for doxycycline, but all the isolates were sensitive to chloramphenicol and trimethoprim-sulfamethoxazole (Table 8). It was evident that through the entire production cycle all the isolates were sensitive to chloramphenicol and were resistant to doxycycline.

In RMS, also highest resistance was recorded against doxycycline (97.22%), and least resistance was found to cefotaxime + clavulanic acid (5.56%). However, isolates were found to be more sensitive to chloramphenicol, colistin sulphate, cefotaxime, ceftazidime, amikacin, amoxicillin + clavulanic acid, ampicillin, and trimethoprim-sulfamethoxazole (Table 7).

### 3.5. ESBL Producers

In the present study, it was observed that 18/106 (16.98%) of the isolates were ESBL producers. The prevalence was 27.7% (10/36) in RMS and 13.59% (8/58) in CBF, and none of the isolates from hatcheries were ESBL producers (Table 7). With respect to the *Salmonella* isolated from CBF, the highest number of ESBL producing isolates was detected on day zero (4/28; 14.29%) followed by days 35-42 (3/20; 15%) and lowest being recorded on days 18-20 (1/10; 10%) (Table 8).

### 3.6. Colistin Resistance

Initially, all the 106 isolates were subjected to disc diffusion assay, and 47 isolates which were found to be resistant were subjected to MIC for colistin. It was observed that in the complete broiler production chain, 40.42% of the isolates were resistant (MIC of >2 *μ*g/mL). No significant difference was observed in colistin resistance with respect to isolates obtained from CBF and RMS, and none of the isolates from hatcheries were resistant to colistin sulphate (Figure 1 and Table 7).

### 3.7. Multidrug Resistance (MDR)

In the present study, it was observed that 76.41% of *Salmonella* isolates were MDR, as defined by resistance to at least one agent in three or more categories of antibiotics [17]. The prevalence of MDR was highest in RMS (91.66%) followed by CBF (82.75%), and none of the hatcheries' isolates exhibited MDR.

### 3.8. AMR Pattern in Complete Supply Chain

In the present study, 100% of the isolates were resistant to at least one antibiotic. Irrespective of the sample source, highest resistance was observed to doxycycline (94.34%) followed by cefpodoxime (84.91%), ciprofloxacin (72.64%), gentamicin (65.09%), enrofloxacin (61.32%), colistin sulphate (40.42%), amikacin (34.91%), ampicillin (33.96%), neomycin (33.02), cefotaxime (30.19%), ceftazidime (29.25%), trimethoprim-sulfamethoxazole (23.58%), amoxicillin + clavulanic acid (21.70%), and chloramphenicol (12.26%) (Table 7).

## 4. Discussion

*Salmonella* is one of the leading foodborne pathogens in humans. Prevalence of *Salmonella* in food animals and increasing AMR poses a continuous threat to one health approach.

*Salmonella* also causes major economic losses to poultry industry. Poultry producers face many direct losses from *Salmonella* infections in their flocks. Infections acquired vertically from parents or horizontally in the hatchery can cause growth retardation or even mortality in young chicks. Preventing the transmission of *Salmonella* to progeny or to humans can be expensive for poultry farmers. In the present study, the prevalence of *Salmonella* was investigated in complete poultry supply chain from hatcheries to retail meat shops.

In the present study, three selective agar media, viz., XLD, BGA, and XLT4, were compared for isolation and recovery of *Salmonella* from various poultry samples, and results revealed that they did not show any significant differences. These findings are in agreement with the earlier findings [18]. However, few researchers [19, 20] have reported that XLT4 was a better medium for isolation of *Salmonella* with nearly 100% success. On the other hand, the colonies not confirmed as *Salmonella* was observed for XLD (143/182) followed by BGA (115/182) and XLT4 (106/182). This may be because of the presence of Tergtitol 4 detergent in XLT4, and this could have inhibited the growth of *Proteus* spp. that produce colonies similar to *Salmonella* in XLD. The comparison of enrichment culture-based PCR assay had detected very higher number of positive samples compared to culture methods. It was evident from this study that enrichment followed by direct DNA extraction from enriched culture and PCR was a sensitive method which eliminates the processing of negative samples. It is also clear that enrichment in TTB followed by selective plating and colony PCR employing both standard culture method in combination with molecular methods such as colony PCR for *Salmonella* detection could be simple, rapid, and effective. This finding was in agreement with earlier studies [21–23].

The overall prevalence of *Salmonella* in the present study was 25.82%. These findings are in line with other researchers who have reported higher prevalence of *Salmonellae* in chilled chicken meat samples (51%) [24], CBF (32.5%), backyard chicken (21.4%) [25], CBF supply chain (14.52%) [26], poultry meat (8.18%) [27], and egg contents (0.5%) [28].

This study revealed varied prevalence of *Salmonella* in different stages of the poultry supply chain including hatcheries (10.34%), CBF (19.19%), and RMS (46.29%). This indicated a gradual increase in the presence of *Salmonella* in the poultry supply chain. Statistical analysis revealed significantly higher risk of *Salmonella* contamination in retail meat shops. Similar findings have been reported earlier [26], where it was shown that majority of the *Salmonella* Enteritidis strains spread along the broiler chicken supply chain. Presence of *Salmonella* in hatcheries is a major point of risk for the complete supply chain such as CBF and even retail meat shops as it causes huge mortality in young chicks and its control becomes very difficult in the farm. In our study, the detection of *Salmonella* in hatcheries (10.34%) was higher as compared to the previous studies with observation of 2.9% in eggs, 2.4% in egg shell, and 0.5% in egg contents [28]. In the present investigation, it was observed that incubator air tunnel had higher presence of *Salmonella*. This is of significance as the entire incubator air is supplied through it, and it could be a source of infection for all the eggs in the incubator. Besides, the presence of *Salmonella* in hatcheries indicated that the hatcheries and its environment have not properly been cleaned and disinfected. Hence, maintenance of cleanliness and hygienic practices during the incubation and hatching process is very important and crucial for control of *Salmonella* infections in chicks and to prevent further transmission of infection to the CBFs.

Similarly, high presence of *Salmonella* (19.19%) was observed in CBF especially at day zero, clearly indicating lack of biosecurity and hygienic practices in CBF. The findings are in concurrence with earlier studies [29–31], where 4.35% to 20% prevalence of *Salmonella* was reported in CBF, respectively. In this study, boot sock samples were collected from the farm environment before arrival of the chicks in the farm, and it was found that such samples were positive for *Salmonella*, indicating that some of the farms were contaminated even before placement of the day-old chicks.

In the present study, the overall prevalence of *Salmonella* in RMS was 46.29%, which is in agreement with the earlier findings of 51% prevalence in India [24] but higher than those reported in chicken meat samples in Turkey (10.64%) [32], broiler supply chain in Qingdao City, China (14.98%) [26], chicken meat from wet markets in Malaysia (20.8%) [33], broiler supply chain in Korea (16.06%) [34], or from different chicken samples in slaughter houses in China (30.14%) [35]. The higher prevalence of *Salmonella* in RMS in our study may be attributed to the fact that majority of the retail meat shops are open type shops, selling meat to the consumers directly, after slaughter of live birds. Moreover, all the operations of bleeding, skinning, and cutting are carried out in small premises without any demarcation of different slaughter and dressing operations. The hygienic practices and cleanliness are at very minimum level in these shops, and on other hand, they receive potentially infected live birds on daily basis from different broiler suppliers. In the present study, prevalence of *Salmonella* in ileal and cecal contents, carcass rinse, meat rinsing water, and knife swabs had indicated that *Salmonella* is distributed among the various samples of the retail meat shops and its environment.

In this study, very high prevalence (65.09%) of *Salmonella enterica* was detected among the poultry isolates. *Salmonella enterica* serotypes are among the most common cause of food poisoning in humans [36]. *Salmonella enterica* represents the most pathogenic species and includes >2600 serovars. *Salmonella* can be transmitted to humans along the farm-to-fork continuum [37]. We found that the prevalence of *Salmonella enterica* was higher in isolates from RMS samples, followed by hatcheries and CBF. Similar finding was reported in China [38], Egypt [39], and Haryana, India [40].

The results of antimicrobial susceptibility testing revealed that 100% of the isolates were resistant to at least one of the antibiotics with 76.41% being MDR. The MDR was highest in RMS followed by CBF and hatcheries. Similar to the findings of our study, high MDR *Salmonella* (100%) was reported from retail chicken meat shops in North India (100%) [30], along the slaughtering line in China (78.6%) [35] and Egypt (76.7%) [39], healthy chicken samples in Korea (63.6%) [41], and poultry isolates in Turkey (46.4%) [32].

The findings of this study clearly indicate that there was higher AMR to tetracyclines, *β*-lactams, fluoroquinolones, aminoglycosides, polymyxin, ESBL inhibitors, folate pathway inhibitors, and less resistance to phenicols. Similar finding has been reported elsewhere for *β*-lactam and macrolide antibiotics (52.9-100%) [25] while lesser resistance to chloramphenicol (3.13%) and higher resistance to ciprofloxacin and *β*-lactams (68.75-100%), tetracycline (65.62%), and colistin sulphate (46.87%) were reported in other studies [42]. Previous researchers have reported that there was shift in antibiotics selection used for treatment of *Salmonella* infections in poultry, from chloramphenicol and ampicillin to trimethoprim-sulfamethoxazole, fluoroquinolones, and extended-spectrum cephalosporins [43, 44]. The unjudicial use of antibiotics may be one of the main reasons for increased resistance to *β*-lactams, tetracycline, and fluoroquinolones.

High prevalence of MDR in Gram-negative bacteria has increased the importance of polymyxins, especially polymyxin E (colistin) for the management of Gram-negative infections in many countries. In the present study, surprisingly, a high prevalence of colistin resistance was observed in entire broiler supply chain (40.42%) although its use is banned in food animals including poultry in India [45] [46]. Similar findings (46.87%) were reported by earlier researchers in India [43]. But higher resistance against colistin (92.68%) was recorded in Bangladesh [47] and lesser (20%) in Serbia [48] and (4.76%) in Mumbai, India [49]. It is beyond the scope of this study to attribute colistin resistance to a particular reason.

Comparison of different segments of the poultry supply chain revealed higher AMR in retail meat shops followed by CBF and hatcheries. This may be due to the frequent use of antibiotics in the CBF and horizontal gene transfer in the intestine of poultry chicken [25]. Cages, workers' hand, and vehicles used for transportation of the chicken may also play a role in transmission of resistant bacteria from farm to retail shops.

## 5. Conclusion

The results of the present study indicated that XLT4 and BGA were found to be specific for isolation followed by colony PCR for identification of *Salmonella* from poultry samples. In addition, enrichment of poultry samples in TTB followed by PCR was found to reduce the preenrichment and isolation protocols. The study also revealed higher prevalence of antimicrobial resistant *Salmonella* in the entire broiler supply chain, which warrants immediate action in terms of reducing the use of antimicrobials as well as biosecurity measures which would help to decrease the emergence of AMR *Salmonella*.

## Figures and Tables

**Table 1:** Samples collected from different commercial broiler supply chain.

No.	Types of samples collected	*n*
Hatchery		29
1	Swabs from egg setting room (10 swabs from different areas and pooled to one/hatchery)	3
2	Swabs from incubator/setter (3 swabs from different areas of each incubator and 50% of the incubator present in each hatchery were sampled and pooled)	3
3	Swabs from air tunnels and fans of incubators/setter (3 swabs from different areas of each incubator and 50% of the incubator present in each hatchery were sampled and pooled)	3
4	Swabs from hatchers (3 swabs from different areas of each hatcher and samples were pooled)	3
5	Swabs from hatchers egg tray (ten trays/hatcher and one swab/tray from different areas and samples were pooled)	3
6	Meconium swabs (ten trays/hatcher and one swab/tray from different areas and samples were pooled)	3
7	Yolk sac swab of dead chicks (ten dead chicks and samples were pooled)	3
8	Hand swabs from hatchery workers (two swabs from two workers)	5
9	Boot socks from hatchery floor	3
Commercial broiler farm (CBF)—three samplings—day 1, days 18-20, and days 35-42		99
1	25 ml water from water tank/shed (25 ml in 25 ml 2x BPW)	15
2	25 ml water from 30 different nipples/shed/(25 ml in 25 ml 2x BPW) and pooled	15
3	25 g feed sample from 10 different feed bags/shed and pooled	15
4	25 g feed sample from 30 different feeders/shed and pooled	15
5	Faecal swabs (30 swabs/shed pooled)	15
6	Internal (inside the shed) environment samples using sterile boot socks/one pair/shed	15
7	External (outside the shed) environment samples using sterile boot socks/farm	9
Retail meat shops (RMS)		54
1	Swabs from surface of cutting/chopping board (100 cm^2^). Swabs were immersed in sterile BPW	3
2	Swabs from cutter/knife	3
3	Meat rinsing water (25 ml in 25 ml 2x BPW)	3
4	Chicken carcasses (5 carcasses/shop)	15
5	Ileal contents from five carcasses	15
6	Cecal contents from five carcasses	15

**Table 2:** Comparison of conventional culturing method and PCR for detection of *Salmonella* in different poultry samples.

No.	Source of sample	Samples collected	XLD		XLT4		BGA		Genomic DNA + for *hilA* gene from TTB culture
			Suspicious growth	Sample tested positive by PCR	Suspicious growth	Sample tested positive by PCR	Suspicious growth	Sample tested positive by PCR	
1	Hatchery	29	20	3	14	0	19	0	4
2	CBF	99	77	14	51	9	59	11	22
3	RMS	54	46	12	41	19	37	18	26
Grand total		182	143	29	106	28	115	29	52^∗∗^

**Table 3:** Detection of *Salmonella* in hatcheries' samples.

No.	Types of samples collected	Total no. of samples	*Salmonella* positive samples
1	Swabs from egg setting room	3	0
2	Swabs from incubator/setter	3	0
3	Swabs from air tunnels and fans of incubators/setter	3	1
4	Swabs from hatchers	3	0
5	Swabs from hatcher egg tray	3	1
6	Meconium swabs	3	0
7	Yolk sac swab of dead chicks	3	1
8	Hand swabs from hatchery workers	5	0
9	Boot socks from hatchery floor	3	0
Total		29	3/29 (10.34%)

**Table 4:** Detection of *Salmonella* in CBF samples.

No.	Type of samples collected	Sampling time points	Total no. of samples	*Salmonella* positive samples
1	Boot socks from internal environment	Day 1, days 18-20, and days 35-42	15	6
2	Faecal swabs		15	5
3	Boot socks from farm external environment		9	3
4	Feed sample from different feeders		15	2
5	Water sample from nipples/drinkers		15	2
6	Feed sample from feed bags		15	1
7	Water sample from water tank		15	0
Total			99	19/99 (19.19%)

**Table 5:** Detection of *Salmonella* in samples collected from RMS.

Type of samples	No. of samples	*Salmonella* positive samples
Ileal contents	15	9
Cecal contents	15	9
Chicken carcasses	15	4
Meat rinsing water	3	2
Swabs from cutter/knife	3	1
Swabs from of cutting/chopping board	3	0
Total number	54	25/54 (46.29%)

**Table 6:** Detection of *Salmonella enterica* in poultry supply chain.

No.	Source of sample	No. of samples collected	No. of *Salmonella* isolates	No. of *Salmonella enterica* isolates
1	Hatcheries	29	12	8
2	CBF	99	58	34
3	RMS	54	36	27
Total		182	106	69/106 (65.06%)

**Table 7:** Antimicrobial resistance of *Salmonella* isolates to various antibiotics.

Class of antibiotic	Name of antibiotics	Percentage of AMR of *Salmonella* isolates				*P* value
		Hatcheries *n* = 12 (%)	CBF *n* = 58 (%)	RMS *n* = 36 (%)	Total *n* = 106 (%)	
Aminoglycosides	GEN	1/12 (8.33)	48/58 (82.76)	20/36 (55.56)	69/106 (65.09)	<0.01
	AK	0/12 (0.00)	23/58 (39.66)	14/36 (38.89)	37/106 (34.91)	<0.02
	N	0/12 (0.00)	18/58 (31.03)	17/36 (47.22)	35/106 (33.02)	<0.01
Fluoroquinolones	CIP	7/12 (58.33)	41/58 (70.69)	29/36 (80.56)	77/106 (72.64)	<0.20
	EX	0/12 (0.00)	38/58 (65.52)	27/36 (75.00)	65/106 (61.32)	<0.01
Tetracycline	DO	7/12 (58.33)	58/58 (100.00)	35/36 (97.22)	100/106 (94.34)	<0.01
Folate pathway inhibitors	COT	0/12 (0.00)	9/58 (15.52)	16/36 (44.44)	25/106 (23.58)	<0.01
Phenicols	C	0/12 (0.00)	2/58 (3.45)	11/36 (30.56)	13/106 (12.26)	<0.01
Penicillin/b-lactamase inhibitors	AMP	0/12 (0.00)	21/58 (36.21)	15/36 (41.67)	36/106 (33.96)	<0.02
	AMC	0/12 (0.00)	9/58 (15.52)	14/36 (38.89)	23/106 (21.70)	<0.01
Polymyxins	CS	0/12 (0.00)	9/28 (32.14)	6/19 (31.58)	19/47 (40.42)	<0.01
Extended-spectrum cephalosporins; 3rd and 4th generation cephalosporins/b-lactamase inhibitors	CTX	1/12 (8.33)	18/58 (31.03)	13/36 (36.11)	32/106 (30.19)	<0.05
	CAZ	0/12 (0.00)	18/58 (31.03)	13/36 (36.11)	31/106 (29.25)	<0.05
	CPD	100.00	45/58 (77.59)	33/36 (91.67)	90/106 (84.91)	<0.01
	CEC	0.00	3/58 (5.17)	2/36 (5.56)	5/106 (4.72)	<0.70
	CAC	0.00	3/58 (5.17)	6/36 (16.67)	9/106 (8.49)	<0.08
	CCL	0.00	2/58 (3.45)	4/36 (11.11)	6/106 (5.66)	<0.19
Average ESBLS		0.00	13.79	27.78	16.98	

**Table 8:** Antimicrobial resistance of *Salmonella* isolates to various antibiotics in different points of CBF crop cycle.

Class of antibiotic	Name of antibiotics	Percentage of AMR of *Salmonella* isolates			*P* value
		Day 0 *n* = 28 (%)	Days 18-20 *n* = 10 (%)	Days 35-42 *n* = 20 (%)	
Aminoglycosides	GEN	23/28 (82.14)	10/10 (100.00)	15/20 (75.00)	<0.02
	AK	8/28 (28.57)	7 (70.00)	8/20 (40.00)	<0.07
	N	2/28 (7.14)	7 (70.00)	3/20 (15.00)	<0.01
Fluoroquinolones	CIP	19/28 (67.86)	10/10 (100.00)	12/20 (60.00)	<0.06
	EX	18/28 (64.85)	10/10 (100.00)	10/20 (50.00)	<0.02
Tetracycline	DO	28/28 (100.00)	10/10 (100.00)	20/20 (100.00)	<0.05
Folate pathway inhibitors	COT	7/28 (25.00)	0/10 (0.00)	10/20 (10.00)	<0.01
Phenicols	C	1/28 (3.57)	0/10 (0.00)	1/20 (5.00)	<0.70
Penicillin/b-lactamase inhibitors	AMP	8/28 (28.57)	4/10 (40.00)	9/20 (45.00)	<0.01
	AMC	3/28 (10.71)	2/10 (20.00)	4/20 (20.00)	<0.62
Polymyxins	CS	6/14 (42.86)	1/6 (16.67)	2/8 (25.00)	<0.18
Extended-spectrum cephalosporins; 3rd and 4th generation cephalosporins/b-lactamase inhibitors	CTX	2/28 (7.14)	7/10 (70.00)	9/20 (45.00)	<0.05
	CAZ	2/28 (7.14)	7/10 (70.00)	9/20 (45.00)	<0.01
	CPD	23/28 (82.14)	9/10 (90.00)	13/20 (65.00)	<0.34
	CEC	2/28 (7.14)	1/10 (10.00)	0/20 (0.00)	<0.40
	CAC	1/28 (3.57)	0/10 (0.00)	3/20 (15.00)	<0. 15
	CCL	4/28 (7.14)	0/10 (0.00)	0/20 (0.00)	<0.10
Average ESBLS		14.29	10.00	15.00	

## Data Availability

Data was analysed using the GraphPad Prism 5 software and Chi-square test.
